# Solitary nodular pulmonary amyloidosis mimicking primary lung cancer: A case report

**DOI:** 10.1016/j.radcr.2026.08.079

**Published:** 2026-09-15

**Authors:** Ryo Hashimoto, Tomoki Nakagawa, Yutaka Kurebayashi, Ryota Masuda

**Affiliations:** a Department of General Thoracic Surgery, Shizuoka City Shimizu Hospital, Shimizu, Japan; b Department of General Thoracic Surgery, Tokai University Hachioji Hospital, Hachioji, Japan; c Department of Diagnostic Pathology, Shizuoka City Shimizu Hospital, Shimizu, Japan; d Division of General Thoracic Surgery, Department of Surgery, Tokai University School of Medicine, Isehara, Japan

**Keywords:** Nodular pulmonary amyloidosis, Solitary pulmonary nodule, Spiculation, FDG-PET/CT, Surgical resection

## Abstract

Nodular pulmonary amyloidosis is a rare, localized form of amyloid deposition in the lung and an uncommon cause of solitary pulmonary nodules. Its radiological features may closely mimic that of primary lung cancer if spiculation or fluorodeoxyglucose uptake is present, making preoperative diagnosis challenging. Here, we report the case of a woman in her 60s who was referred to our hospital after an abnormal opacity was detected by routine chest radiograph. Chest computed tomography revealed a 21 mm × 12 mm solid nodule with partial spiculation in the left lower lobe. Fluorodeoxyglucose positron emission tomography/computed tomography demonstrated mild uptake (maximum standardized uptake value; 2.5). Bronchoscopic biopsy results were inconclusive, and diagnostic video-assisted thoracoscopic left basal segmentectomy was performed because malignancy could not be excluded. Histopathological examination under polarized light microscopy demonstrated Congo red-positive amyloid deposition with apple-green birefringence, confirming nodular pulmonary amyloidosis. This case highlights that nodular pulmonary amyloidosis can mimic primary lung cancer on imaging and diagnostic surgical resection may provide definitive diagnosis and local treatment if bronchoscopic biopsy is inconclusive. Long-term follow-up is warranted, considering its reported association with lymphoproliferative disorders.

## Introduction

Pulmonary amyloidosis is a rare disorder characterized by extracellular deposition of amyloid proteins within the lungs. It is classified into nodular pulmonary, diffuse alveolar septal, and tracheobronchial amyloidosis [1,2]. Among these, nodular pulmonary amyloidosis is the most common localized form; however, it remains an uncommon cause of solitary pulmonary nodules.

Nodular pulmonary amyloidosis may closely mimic primary lung cancer or metastatic tumors. Spiculated margins on computed tomography (CT) and uptake on fluorodeoxyglucose positron emission tomography/CT (FDG-PET/CT) have been reported, making preoperative diagnosis challenging [[3], [4], [5]]. Here, we report a case of solitary nodular pulmonary amyloidosis that mimicked primary lung cancer and was definitively diagnosed by diagnostic surgical resection.

## Case presentation

A woman in her 60s was referred to our hospital after chest radiograph detected an abnormal opacity during a routine health checkup. She was asymptomatic and denied coughing, sputum production, hemoptysis, fever, chest pain, or weight loss. Her medical history included a previous episode of left pneumothorax, resection of a retroauricular exostosis, hypertension, dyslipidemia, and depression. She had never smoked. Her family history was unremarkable for amyloidosis, hematologic malignancy, autoimmune diseases, or lung cancer.

The initial laboratory tests revealed no clinically significant abnormalities. The white blood cell count was 3700/µL (reference range [RR]; 3500-9000/µL), hemoglobin was 11.8 g/dL (RR; 11.0-16.0 g/dL), platelet count was 208 × 10^3^/µL (RR; 150-350 × 10^3^/µL), C-reactive protein was 0.02 mg/dL (RR; 0.00-0.30 mg/dL), serum creatinine was 0.58 mg/dL (RR; 0.40-1.20 mg/dL), and estimated glomerular filtration rate was 78 mL/min/1.73 m^2^. Urinalysis showed no proteinuria. Tumor markers were normal, including carcinoembryonic antigen 2.8 ng/mL (RR; <5.0 ng/mL), cytokeratin-19 fragment 1.0 ng/mL (RR; <3.5 ng/mL), and pro-gastrin-releasing peptide 61.1 pg/mL (RR; <81.0 pg/mL).

**Fig. 1:**
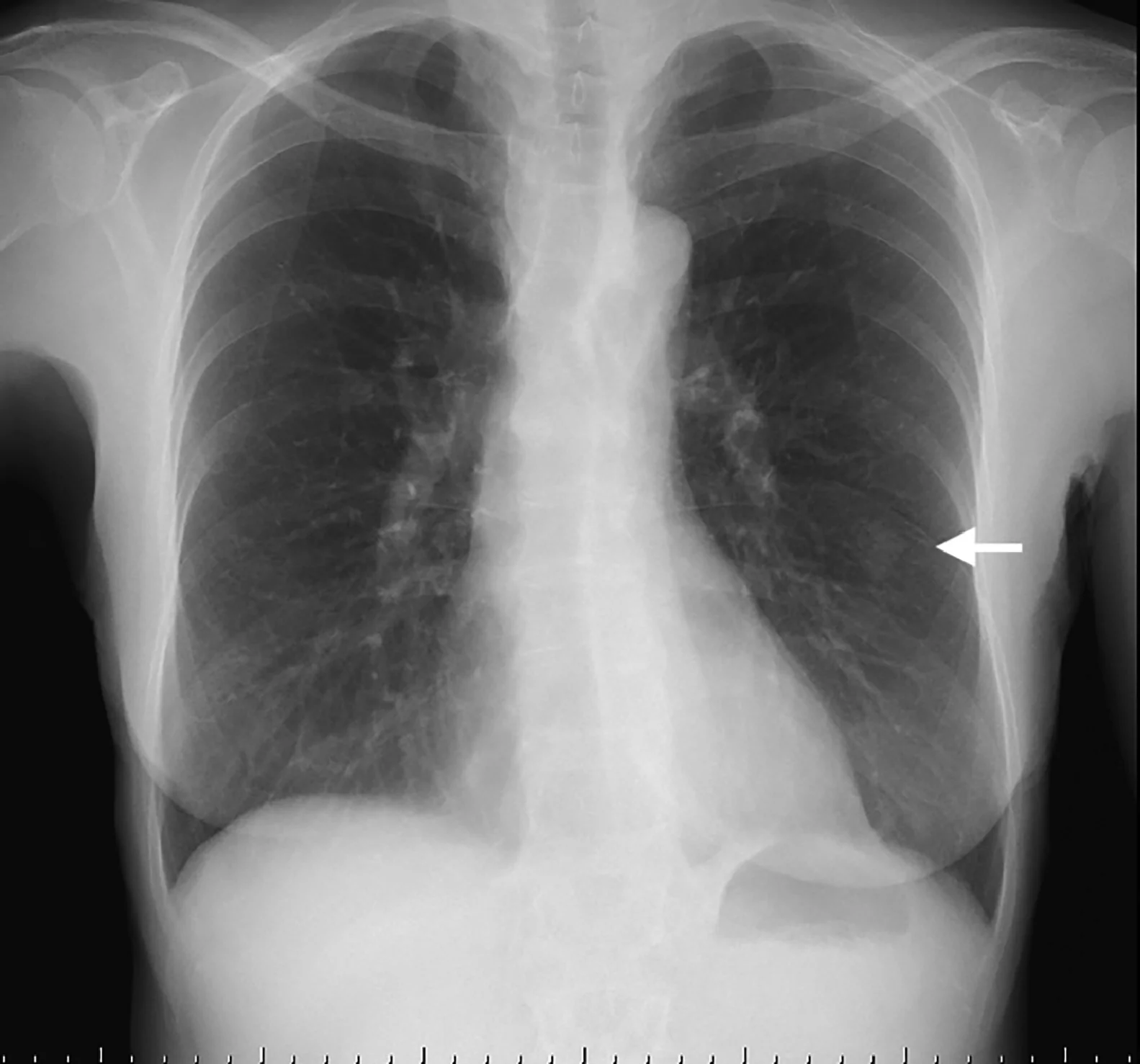
Chest radiograph reveals a nodular opacity in the left lower lung field (arrow), without obvious calcification or cavitation, prompting further evaluation with computed tomography.

**Fig. 2:**
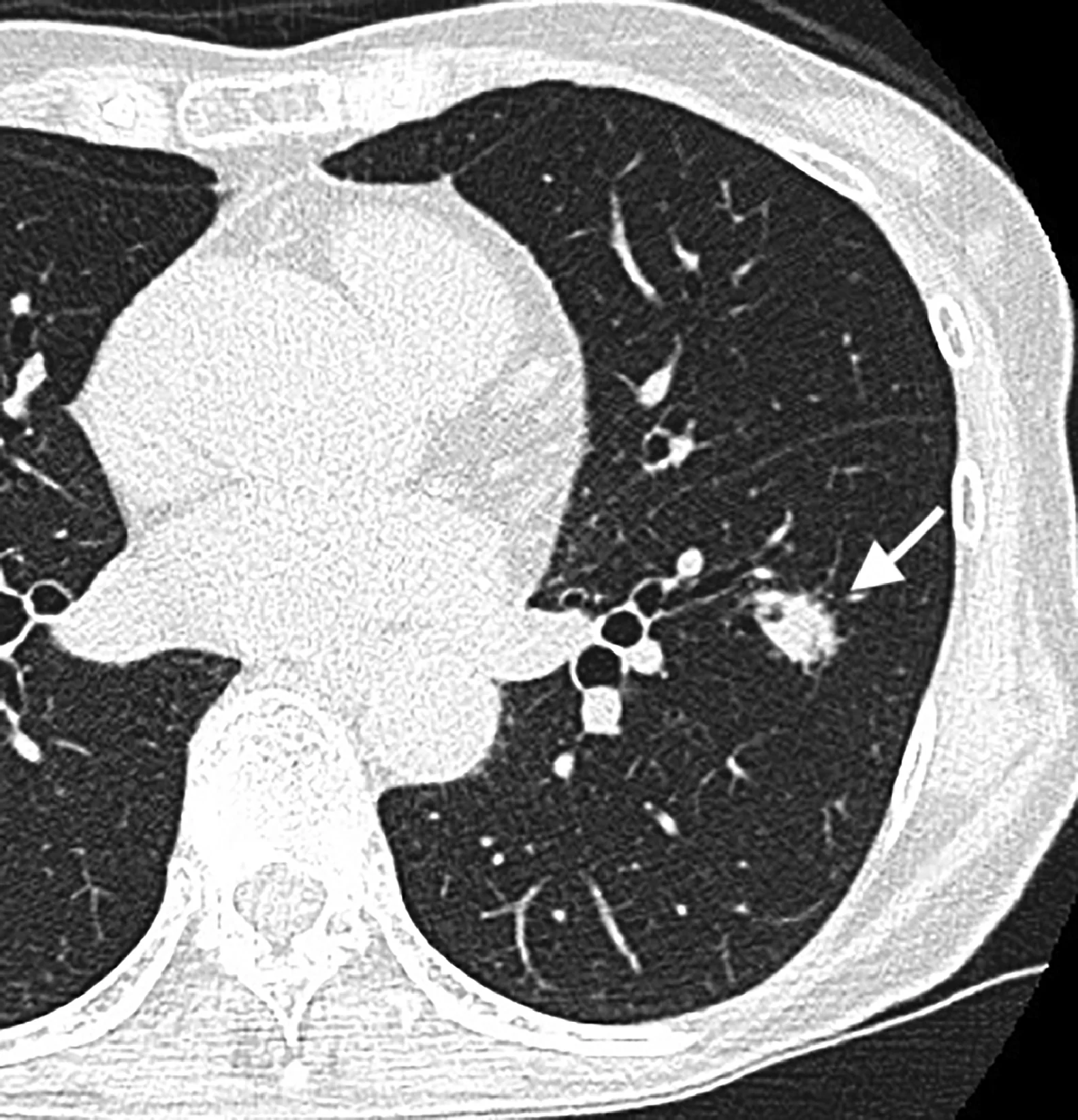
Chest computed tomography (lung window setting) demonstrates a 21 mm × 12 mm solid nodule in segment 10 of the left lower lobe (arrow). Although the margin is relatively well defined, partial spiculation is observed, raising suspicion for primary lung cancer.

Chest radiography revealed a nodular opacity in the left lower lung field (Fig. 1). Chest CT demonstrated a 21 mm × 12 mm solid nodule in segment 10 of the left lower lobe (Fig. 2). Although the nodule margin was relatively well-defined, partial spiculation was observed. No obvious calcification, cavitation, cystic changes, pleural effusion, or mediastinal lymphadenopathy was observed. No apparent growth was observed compared with CT obtained 3 months previously; however, the short interval of stability was insufficient to establish benignity because of the spiculated morphology.

**Fig. 3:**
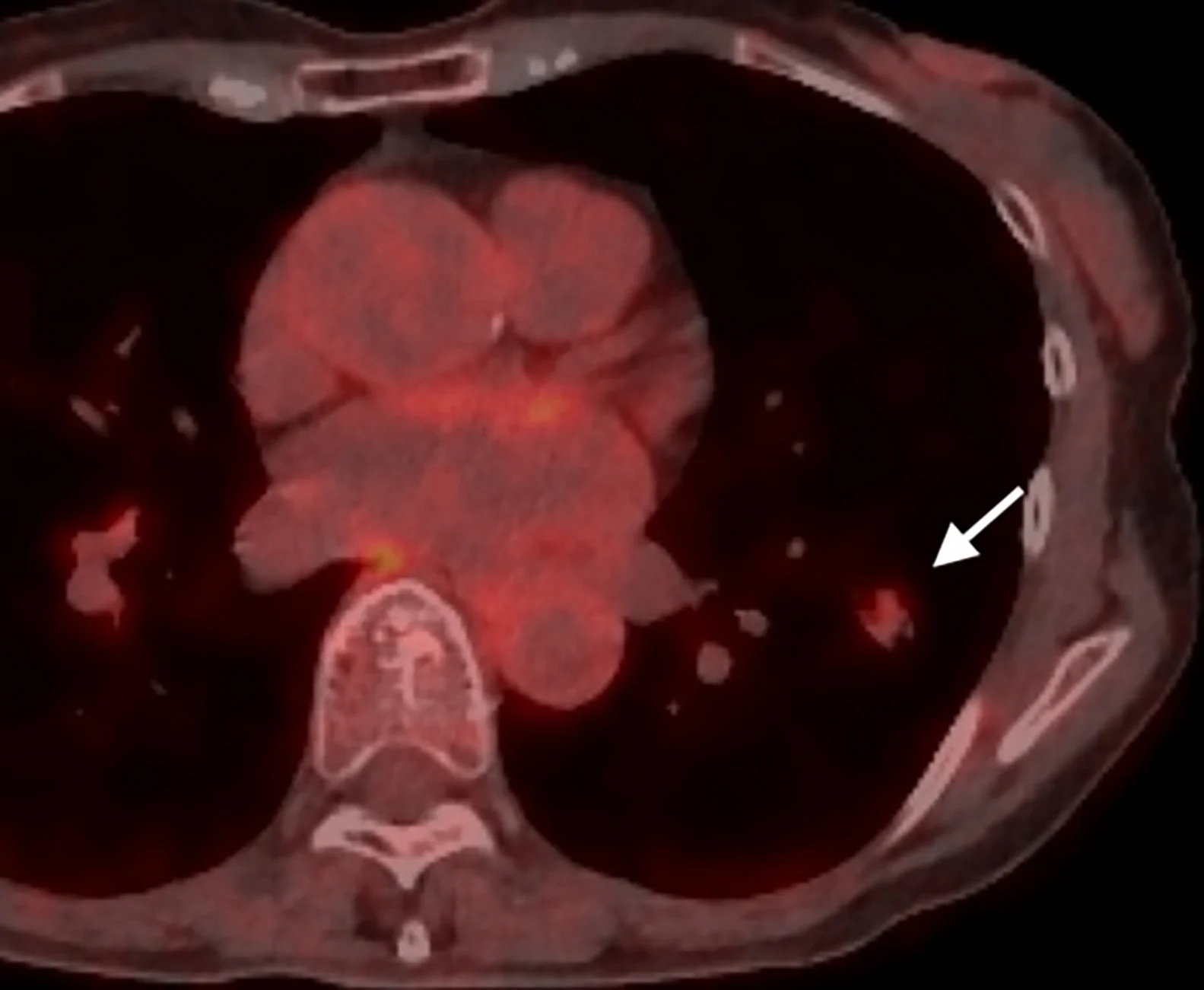
Fluorodeoxyglucose positron emission tomography/computed tomography demonstrates mild FDG uptake corresponding to the pulmonary nodule (arrow), with a maximum standardized uptake value of 2.5. Malignancy could not be excluded based on this finding alone.

FDG-PET/CT revealed mild FDG uptake corresponding to the pulmonary nodule (maximum standardized uptake value; 2.5, Fig. 3). The mean standardized uptake value of the mediastinal blood pool was not documented in the original report and could not be measured retrospectively from the available FDG-PET/CT images. Bronchoscopic biopsy was performed; however, the tissue obtained was insufficient for a definitive diagnosis. CT-guided percutaneous biopsy was not performed because the nodule was located relatively deep in the lung parenchyma, and the risk of procedure-related pneumothorax was considered. Because primary lung cancer remained a concern after nondiagnostic bronchoscopy and the lesion was considered surgically resectable, diagnostic surgical resection was selected to obtain sufficient tissue for definitive diagnosis and local treatment.

The patient underwent video-assisted thoracoscopic left basal segmentectomy. Segmentectomy was selected because the nodule was located in the basal segment of the left lower lobe and sufficient tissue volume and surgical margins were required in cases in which the lesion represented primary lung cancer. Intraoperative frozen section analysis revealed no malignancy; therefore, no additional resection or lymph node dissection was performed. The operation time was 103 minutes, and blood loss was minimal.

**Fig. 4:**
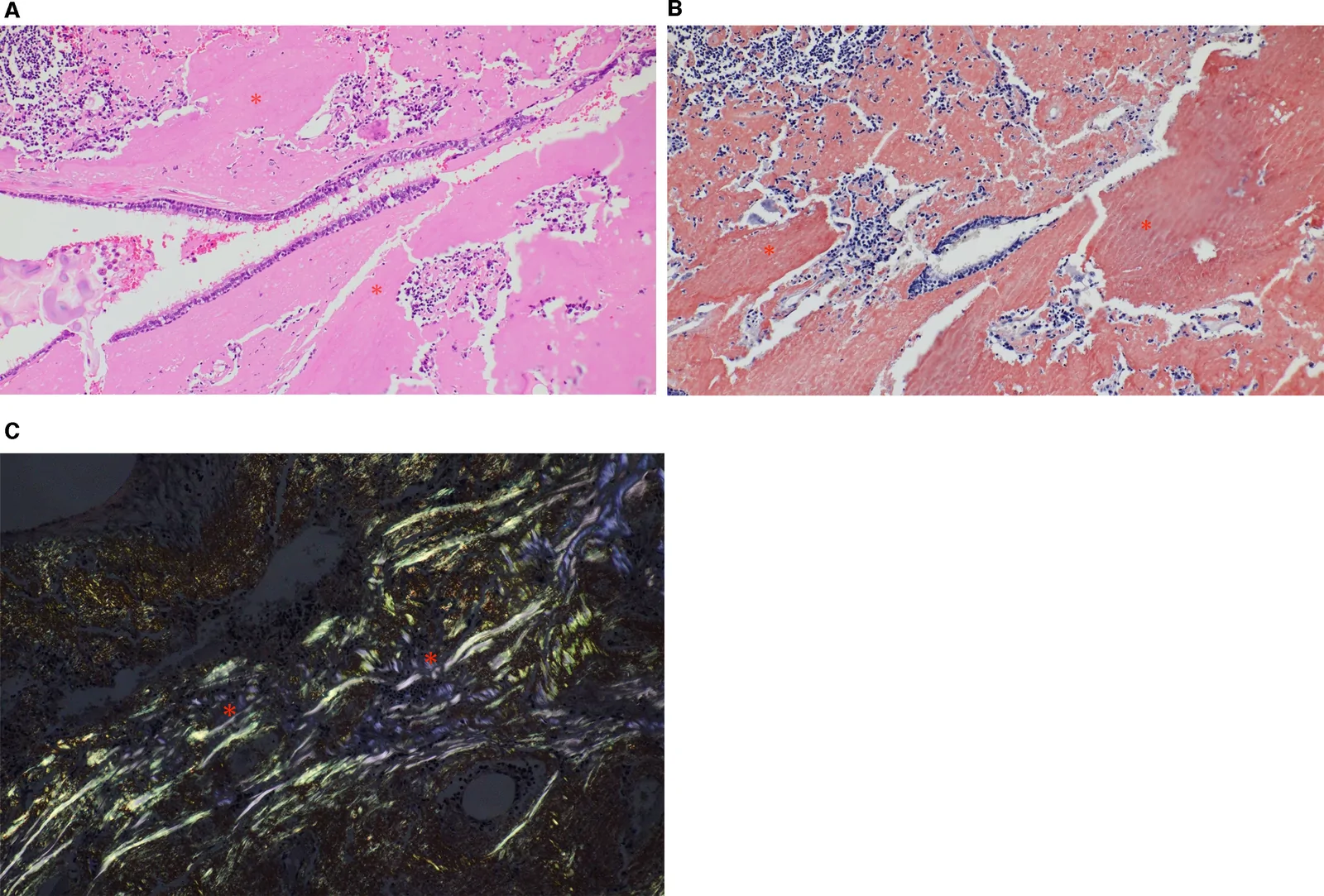
Histopathological findings. Asterisks indicate representative areas of amyloid deposition. (A) Hematoxylin and eosin staining shows deposition of eosinophilic amorphous material within the lung parenchyma (×100). (B) The deposited material is positive on Congo red staining (×100). (C) Polarized light microscopy demonstrates characteristic apple-green birefringence (×100), findings consistent with nodular pulmonary amyloidosis.

Histopathological examination of the resected specimen demonstrated the deposition of eosinophilic amorphous material within the lung parenchyma on hematoxylin and eosin staining (Fig. 4A). The deposited material was positive for Congo red staining (Fig. 4B) and demonstrated the characteristic apple-green birefringence under a polarized light microscope (Fig. 4C), confirming amyloid deposition. No atypical lymphoid proliferation suggestive of a lymphoma was observed. These findings were consistent with those of nodular pulmonary amyloidosis.

The postoperative course was uneventful, and the patient was discharged on postoperative day 6. The available systemic evaluations revealed no evidence of systemic amyloidosis. Renal function was normal, urinalysis revealed no proteinuria, and echocardiography revealed no findings suggestive of cardiac amyloid involvement. Serum and urine protein electrophoreses and immunofixation were not performed. Approximately 24 months postoperatively, the patient remained well. Follow-up chest CT performed at 6-month intervals showed no evidence of local recurrence or new pulmonary nodules.

## Discussion

Nodular pulmonary amyloidosis is a localized form of amyloid deposition that is often detected incidentally [1,2]; however, their radiological appearance may closely mimic malignant pulmonary nodules, resulting in significant diagnostic difficulties. The suspicious CT features of pulmonary nodules include spiculation and irregular margins [6]. In this case, the partial spiculation of the nodule strongly suggested malignancy. Although there was no apparent growth over a 3-month period, this short stability interval was insufficient to establish benignity in the spiculated solid nodule, supporting the decision to proceed with surgical resection.

Besides spiculation, recognized CT features of nodular pulmonary amyloidosis include calcification, lobulation, multiplicity, slow growth, long-term stability, and peripheral or subpleural distribution [3,7]. In some cases, nodular pulmonary amyloidosis may be associated with cystic changes [2,7]. Other subtypes of pulmonary amyloidosis must be identified. Diffuse alveolar septal amyloidosis presents with diffuse interstitial abnormalities, such as reticulonodular opacities, interlobular septal thickening, or ground-glass opacity, whereas tracheobronchial amyloidosis may present with nodular or circumferential airway wall thickening, calcified airway lesions, and airway narrowing [1,2,7].

Previous studies have reported that nodular pulmonary amyloidosis may present with lobulated or spiculated margins, mimicking primary lung cancer [3]. Additionally, mild-to-moderate FDG uptake on FDG-PET/CT has been described in patients with nodular pulmonary amyloidosis [4,5]. FDG avidity is associated with a localized inflammatory reaction involving giant and plasma cells surrounding amyloid deposits. Therefore, the discriminatory value of FDG-PET/CT in differentiating this benign entity from malignant ones is limited, and standardized uptake values alone should be interpreted cautiously.

Histological confirmation is essential for diagnosis; however, bronchoscopic biopsy may provide insufficient tissue for a definitive diagnosis, as in this case [8]. Amyloid deposits are frequently located in deeper fibrotic areas of lesions, and superficial biopsy specimens may not capture the diagnostic material.

If malignancy cannot be excluded, diagnostic surgical resection remains a reliable approach to achieve a definitive diagnosis [8]. In this case, segmentectomy provided an adequate volume of tissue with sufficient surgical margins, while preserving lung function.

Complete surgical resection of nodular pulmonary amyloidosis is generally considered curative for localized lesions. Most reported cases of nodular pulmonary amyloidosis are of the AL type. However, as a limitation of this case report, immunohistochemical subtyping of the resected specimen was not performed [2,9]. Furthermore, serum and urine protein electrophoresis and immunofixation were not performed; therefore, underlying monoclonal gammopathy could not be excluded. However, renal, urinary, or cardiac findings suggestive of systemic amyloidosis were not observed. Importantly, nodular pulmonary amyloidosis is associated with B-cell lymphoproliferative disorders, including mucosa-associated lymphoid tissue (MALT) lymphoma [9]. Additionally, chronic autoimmune conditions, such as Sjögren’s syndrome, which is characterized by persistent B cell activation, contributes to this background [2,9]. Therefore, long-term follow-up is recommended to monitor for the potential development of associated lymphoproliferative diseases, even after complete resection [2,9].

## Patient consent

Written informed consent was obtained from the patient for the publication of this case report and any accompanying images. A copy of the written consent is available for review by the Editor-in-Chief of this journal on request.
